# Role of Polyphenols and Carotenoids in Endothelial Dysfunction: An Overview from Classic to Innovative Biomarkers

**DOI:** 10.1155/2020/6381380

**Published:** 2020-10-19

**Authors:** Natalia Di Pietro, Maria Pompea Antonia Baldassarre, Angelo Cichelli, Assunta Pandolfi, Gloria Formoso, Caterina Pipino

**Affiliations:** ^1^Department of Medical, Oral and Biotechnological Sciences, University G. d'Annunzio of Chieti-Pescara, 66100 Chieti, Italy; ^2^Center for Advanced Studies and Technology-CAST (ex CeSI-MeT), University G. d'Annunzio of Chieti-Pescara, 66100 Chieti, Italy; ^3^Department of Medicine and Aging Sciences, University G. d'Annunzio of Chieti-Pescara, 66100 Chieti, Italy

## Abstract

Nowadays, the dramatically increased prevalence of metabolic diseases, such as obesity and diabetes mellitus and their related complications, including endothelial dysfunction and cardiovascular disease, represents one of the leading causes of death worldwide. Dietary nutrients together with healthy lifestyles have a crucial role in the endothelium health-promoting effects. From a growing body of evidence, active natural compounds from food, including polyphenols and carotenoids, have attracted particular attention as a complementary therapy on atherosclerosis and cardiovascular disease, as well as preventive approaches through the attenuation of inflammation and oxidative stress. They mainly act as radical scavengers by promoting a variety of biological mechanisms, such as improvements in endothelial function, blood pressure, platelet activity, and insulin sensitivity, and by modulating various known biomarkers. The present review highlights the role of polyphenols and carotenoids in early endothelial dysfunction with attention to their beneficial effect in modulating both classical and recent technologically generated emerging biomarkers. These, alone or in combination, can play an important role in the prediction, diagnosis, and evolution of cardiovascular disease. However, a main challenge is to speed up early and prompt new interventions in order to prevent or slow down disease progression, even with an adequate intake of bioactive compounds. Hence, there is an urgent need of new more validated, appropriate, and reliable diagnostic and therapeutic biomarkers useful to diagnose endothelial dysfunction at an earlier stage.

## 1. Introduction

### 1.1. Diet Rich in Polyphenols and Carotenoids as Potential Allies for Cardiovascular Health

Among the various dietary patterns that provide for a large consumption of foods containing polyphenols and carotenoids, the Mediterranean diet (MD) is certainly an excellent example [1, 2]. Indeed, MD represents a complete and balanced combination of antioxidant-rich foods with proven protective action against cardiovascular diseases (CVD) [3, 4]. This high-quality dietary pattern is mainly based on plant-derivate foods (fresh fruits, vegetables, legumes, and tree nuts) which represent important sources of bioactive compounds, such as antioxidant vitamins, minerals, and various phytochemicals, and are also the main asset of polyphenols and carotenoids in the human diet [4–7].

Polyphenols are a large and heterogeneous group of phytochemicals generally divided into flavonoids, phenolic acids, stilbenes, and lignans, although more than 8000 different polyphenols have so far been described [8]. These molecules are produced in plants as secondary metabolites to manage environmental stresses, such as ultraviolet lights, free radicals, and uncommon temperatures, thus limiting the effects of oxidative stress. Therefore, their main property is related to their structure which is characterized by the presence of one or more phenolic groups, capable of reducing the reactive oxygen species (ROS) and various organic substrates and minerals [9]. This explains the significant interest in polyphenol role in preventing several chronic diseases associated with oxidative stress [9]. Of note, they have been shown to modulate a variety of targets in the prevention of cardiovascular diseases, which include platelet function, blood pressure, endothelial function, and blood lipids [10].

Another class of antioxidants naturally contained in fruit and vegetables are carotenoids. They are fat-soluble pigments consisting in carbon-40 isoprenoid structure and characterized by the presence of numerous conjugated double bonds which determine their color: the increase in the number of double bonds is associated with changes in color from pale yellow to orange and red, reflecting the colors of the plants and fruits that contain them [11].

To date, more than 700 carotenoids have been characterized; about 50 of them consumed in the human diet, but only about 10-15 are the carotenoids most consumed with food and which have detectable concentrations in human blood and tissues [12, 13]. Among these, *β*-carotene, *α*-carotene, and *β*-cryptoxanthin are the main carotenoids with significant provitamin A activity, while lutein, lycopene, and zeaxanthin are not converted into active retinoids by humans [14]. Several *in vitro* and *in vivo* studies have shown that carotenoids are able to reduce inflammation and oxidative stress through the regulation of various cellular functions, thus supporting the epidemiological studies indicating a strong correlation between dietary carotenoid consumption and decreased risk of CVD [12, 13, 15, 16].

Therefore, based on the explanation above, we can propose that a dietary intervention with foods rich in polyphenols and carotenoids might prevent and/or limit endothelial dysfunction, which is the starting point of the atherosclerotic process and accompanies the progression of CVD [1, 17].

### 1.2. Endothelial Function and Dysfunction

The vascular endothelium, consisting of an endothelial cell (EC) monolayer, extends along the entire circulatory system acting as a semipermeable barrier and ensuring the maintenance of vascular homeostasis. Indeed, ECs through their multiple actions can be considered real sentinels for cardiovascular and metabolic health [18].

ECs interact with circulating cells on the one hand, and cells present in the vascular wall, such as vascular smooth muscle cells (VSMCs), on the other hand. Furthermore, being the interface between blood and tissue, they are affected by changes in blood composition and blood flow. Any alteration of these parameters induces the conversion of the endothelium from an antithrombotic, anti-inflammatory, and vasodilatory phenotype to a procoagulant, inflammatory, and vasoconstrictor phenotype [19].

Under healthy condition, ECs synthesize and release a wide spectrum of vasoactive substances including the nitric oxide (NO) with relevant vasorelaxant and protective functions. The production of this soluble gas is regulated by the endothelial nitric oxide synthase (eNOS), enzymatic isoform constitutively expressed in ECs, through the conversion of L-arginine to L-citrulline and NO [20].

Once synthesized, NO diffuses into the VSMCs where, by stimulating the soluble guanyl cyclase and increasing the cyclic guanosine monophosphate (cGMP, biological effector of NO), it promotes vasodilation. In addition, NO exerts antiproliferative effects on VSMC and inhibits adhesion and aggregation of platelets, as well as leucocyte adhesion and migration in the arterial wall, showing anti-inflammatory properties [21].

However, these positive effects in maintaining endothelial functions can be disturbed by a growing list of conditions, which are also recognized risk factors associated with the onset and progression of endothelial dysfunction and atherosclerosis. These include obesity, hypertension, hypercholesterolemia, insulin resistance, diabetes mellitus, uremia, and heart failure [22–27]. Furthermore, nonmodifiable factors such as aging and genetic as well as modifiable factors like smoking, lifestyle, and eating habits also contribute to vascular endothelial activation.

In these conditions, the EC phenotype turns into a proinflammatory and prothrombotic state, characterized by a reduced bioavailability of NO counterbalanced by an increase in other substances and mediators that are harmful to the arterial wall [17, 28, 29].

Among these, the biological effects induced by endothelin-1 (ET-1) involve vasoconstrictor and proinflammatory actions and its ability to induce the release of proinflammatory cytokines such as interleukin- (IL-) 1, IL-6, and IL-8 [30]. Furthermore, the decrease of NO bioavailability promotes the activation of VSMCs in a synthetic state, which allows them to proliferate and migrate in the intima and to produce an extracellular matrix that leads to the formation of neointima and fibrous component of the atherosclerosis [30].

**Figure 1:**
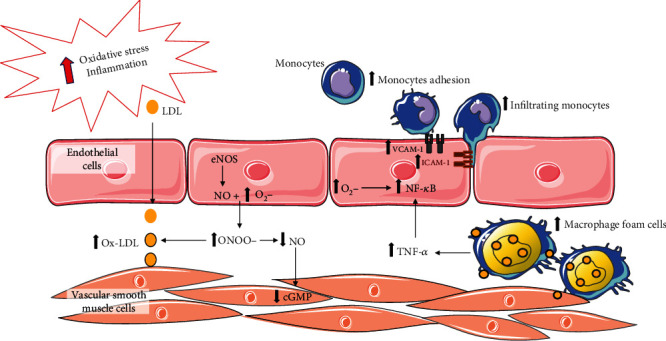
Endothelial dysfunction. *Figure adapted from Di Pietro N. et al. JSM Atherosclerosis, 2016* [12]. Under oxidative condition, NO may react with O_2_^−^ to form ONOO^−^; this leads to the decrease of NO bioavailability leading to endothelial dysfunction, enhanced LDL peroxidation, and chronic vascular inflammation. This is associated with lipid accumulation in the arterial wall, an NF-*κ*B activation that in turn triggers the upregulation of VCAM-1 and ICAM-1. The increased VCAM-1 and ICAM-1 membrane exposure leads to increased adhesion and infiltration of monocytes. eNOS: endothelial nitric oxide synthase; NO: nitric oxide; O_2_^−^: superoxide anion; ONOO^−^: peroxynitrite; cGMP: cyclic guanosine monophosphate; LDL: low-density lipoprotein; ox-LDL: oxidized low-density lipoprotein; TNF-*α*: tumor necrosis factor alpha; NF-*κ*B: nuclear factor kappa-light-chain-enhancer of activated B cells; ICAM-1: intercellular adhesion molecule 1; VCAM-1: vascular cell adhesion molecule 1.

As shown in Figure 1, these dysfunctional endothelial changes are further exacerbated by the coexistence and increase of oxidative stress and inflammation [28]. It is noteworthy that in early stages of atherogenesis, a crosstalk between oxidative stress and inflammation has been proved [28]. On the one hand, ROS contribute in reducing the NO bioavailability through the rapid oxidative inactivation of NO by excess superoxide (O_2_^−^). Indeed, through the reaction between NO and O_2_^−^, more harmful radical species are formed such as peroxynitrite (ONOO^−^), which lead to permanent EC damage with consequent proatherogenic effect. In addition, the tetrahydrobiopterin (BH4) cofactor is highly sensitive to the oxidation of ONOO^−^ and the reduction of BH4 also promotes the production of O_2_^−^ by eNOS (referred to as uncoupling of eNOS) [20]. On the other hand, ROS exert their actions through the nuclear factor kappa-light-chain-enhancer of activated B cells (NF-*κ*B), inducing the synthesis of proinflammatory cytokines, such as tumor necrosis factor-*α* (TNF-*α*), which in turn activate NF-*κ*B [31].

The synergy between ROS and cytokines promotes the synthesis of inflammatory factors in the EC and regulates the expression of adhesion molecules such as intercellular adhesion molecules (ICAM-1) and vascular adhesion molecules (VCAM-1), thus allowing neutrophils to transmigrate inside the vessel wall [32]. Moreover, the increased permeability of ECs allows the accumulation in the tunica intima of circulating low-density lipoproteins (LDLs), which in a prooxidant environment become oxidized LDLs (oxLDLs), more harmful and able of damaging the endothelium and triggering the inflammatory process [33, 34].

All together, these effects are responsible for the initial loss of endothelial functions, which can be prevented and even recovered by removing pathological stimuli and/or improving lifestyle and eating habits.

By focusing on the latter, a growing body of evidence, including *in vitro*, *in vivo* studies and clinical trials discussed below, highlight the key role of nutrition in the maintenance and recovery of endothelial functions [35].

### 1.3. Biomarkers of Vascular Function and Their Potential Modulation by Nutritional Molecules

To date, the scientific community currently highlights the need to identify both reliable instrumental methods and validated circulating biomarkers (i.e., not only putative biomarkers) for early endothelial dysfunction detection [36].

As regards the instrumental methods for the endothelial function determination, the most used for its noninvasive approach is certainly the flow-mediated vasodilation (FMD) of the brachial artery. In addition to this, among the peripheral measurement techniques, we also have the plethysmography of the forearm circulation, the finger plethysmography of the fingers, and the evaluation of the retinal endothelial function, all with specific advantages and disadvantages including the fact that they are operator-dependent and can be influenced by many physiological variations [36].

For these reasons, circulating biomarkers are complementary and promising tools. So far, numerous studies have been conducted considering traditional, also defined “classic”, endothelial circulating markers, including soluble adhesion molecules, such as E-selectin, ICAM-1, and VCAM-1, as well as molecules involved in the coagulation pathway, in particular von Willebrand factor (vWF) and soluble thrombomodulin, and also inflammatory markers such as interleukins IL-6, IL-8, and IL-12 and high-sensitivity C-reactive protein (hsCRP) [37–39].

More recently, extracellular vesicles (EVs) are proving to be potential endothelial dysfunction biomarkers and CVD predictors. EVs are small vesicles (0.1-1.0 mm) released by the plasma membrane of several cells, such as leukocytes, platelets, or ECs. EVs contain biological materials from parental cells, such as adhesion molecules, pro- and anticoagulant substances, and microRNAs, which are released into the circulation and/or transmitted to other types of cells, thus regulating gene expression and influencing cellular pathways [40].

Interestingly, it has been proposed that EVs from endothelial, but not those from other cellular origin, could be very useful for stratifying cardiovascular risk and for monitoring disease activity, severity, and the effectiveness of atheroprotective treatments [37, 40–42].

Furthermore, although diet and nutritional molecules can play an important protective role on cardiovascular health, the way to monitor the process and translate relevant findings in this field is still under investigation [2].

Thus, another ambitious aim would be to identify new food and nutrient intake biomarkers, which could potentially provide new tools for monitoring compliance and evaluating dietary intake in the nutritional and health sciences [43].

More recently, through the development of epigenetics and omics sciences, new promising endothelial dysfunction biomarkers emerged have been identified which could be very useful in measuring the role of nutritional molecules on endothelial function [39, 43–48].

In particular, epigenetic markers (such as noncoding RNA, histone modification, and DNA methylation) that are associated with endothelial dysfunction could represent an important source for monitoring the effectiveness of nutritional molecules on endothelial functions [44, 49–51].

In addition, it would be even more interesting to integrate the omics sciences, including genomics, epigenomics, transcriptomics, and metabolomics, in studies that analyze the impact of nutrition and modulated biomarkers in CVD [48, 52–54]. Indeed, although the benefits of a healthy diet are attributable to its wealth of bioactive compounds, in the era of “Precision Medicine” and “Precision Health”, the new concept of “Precision Nutrition” was recently launched, which refers specifically to the integration of omics markers and personalized diet in order to improve prevention and/or treatment of diseases [48, 53–55].

Based on what has been discussed above, the purpose of this review is to offer a detailed analysis of the state of the art of polyphenols and carotenoids, focusing on their actions on endothelial dysfunction in *in vitro*, *ex vivo*, and *in vivo* animal and human studies, together with a critical review of how these are supported by “validated” measurements both in terms of instrumental diagnostics and traditional and innovative biomarkers.

## 2. Polyphenols

### 2.1. Effects of Polyphenols on the Modulation of Classical Biomarkers of Endothelial Dysfunction: *In Vitro*, *Ex Vivo*, and Experimental Animal Studies

A body of scientific evidences has shown the ability of polyphenols, powerful antioxidant compounds with indicated antiatherogenic properties, to induce endothelium-dependent relaxation and prevent some chronic diseases associated with oxidative stress through modulation of specific biomarkers. As mentioned above, the “classical” biomarkers of endothelial dysfunction may be classified in three main categories: oxidative (e.g., ROS, superoxide anion, and nitrotyrosine), inflammatory (e.g., the soluble adhesion molecules, IL-6, IL-8, IL-12, and hsCRP), and coagulation pathway markers (e.g., vWF, soluble thrombomodulin).

*In vitro*, *ex vivo*, and experimental animal studies have been performed to investigate the potential protective role of polyphenols from food. Most of the *in vitro* studies developed to test the effects of natural bioactive compounds have been performed on ECs, mainly human umbilical vein endothelial cells (HUVECs) that have been considered advantageous to study endothelial function in both normal and diseased conditions [56]. Moreover, some interesting evidences come from data obtained on isolated arterial rings and have indicated that a great variety of polyphenols would cause notable relaxations of precontracted arterial rings [57].

More in detail, polyphenols have been considered effective free radical scavenger mainly acting against elevated production of ROS linked to endothelial dysfunction. It is well known that uncontrolled ROS production alters the vascular tone, which is mediated by reduced NO bioavailability, leading to impaired endothelium-dependent vasodilatation (see Figure 1).

Of note, a considerable group of flavonoids, including those found in cocoa beans, rosmarinic acid, and lemon grass, have been proven to protect against oxidative stress in endothelial dysfunction induced by oxidative stimuli, such as hydrogen peroxide, in both endothelial cells and rat aorta rings, through a NO-independent vasodilator effect [58–60]. Again, polyphenols found in strawberry extract activate the PI3 kinase/Akt pathway in HUVECs, resulting in phosphorylation of eNOS and Akt and induced endothelium-dependent relaxation [61]. Moreover, pretreatment of human aortic endothelial cells (HAECs) with chlorogenic acid (CGA), polyphenols rich in coffee, showed increased endothelial cell viability and NO production in a dose-dependent manner and reduced S-nitrosothiols, nitrite, and nitroso species [62]. Furthermore, the protective effect of CGA on endothelial relaxation was observed in isolated aortic rings from C57BL/6 mice following addition of hypochlorous acid able to induce endothelial dysfunction [62].

In addition, under oxidative stress condition, oxidized lipids may be generated, thus causing vascular damage. The oxidation of LDL leads to conformational changes with formation of foam cells and intracellular lipid retention. Several polyphenols present in pigmented fruits including dark grapes, pomegranates, berries, and some vegetables have been found efficient in attenuating oxLDL-induced mitochondrial dysfunction through reduction of ROS and superoxide anion generation in HUVECs [63].

Interestingly, some polyphenols were found able to decrease advanced glycation end-products (AGEs) accumulation, thus acting as anti-AGE agents potentially preventing endothelial dysfunction [64]. Indeed, the implication of AGEs in endothelial dysfunction is now well known and the consumption of food rich in polyphenols may be advantageous in preventing cardiovascular alterations, thus avoiding clinical events.

Some polyphenols have been found capable of modulating intracellular ROS generation in HUVECs exposed to chronic hypoxia, thus improving the antioxidant cellular activity [65]. Anthocyanin cyanidin-3-O-glucoside (C3G), commonly present in vegetables from the Mediterranean diet and particularly rich in blackberry extract, was found to prevent oxidative stress while improving antioxidant systems and activating the NF-E2-related factor 2/antioxidant responsive element (Nrf2/ARE) pathway with the involvement of specific mitogen-activated protein kinases (MAPKs) (ERK1/2) in HUVECs [66]. Its antioxidant activity was also confirmed in vascular rings exposed to peroxynitrite, thus improving vascular contractility [67].

Besides their role against oxidative stress, the first anti-inflammatory activity of polyphenols in improving endothelial function is the inhibition of lymphocyte and monocyte adhesion to the endothelium, together with the inhibition of the inflammatory mediators. Indeed, it was discovered that luteolin, a bioactive compound mainly found in green vegetables, was able to interfere with the early inflammatory atherogenic process by inhibiting the monocyte-endothelium interactions [68, 69], the monocyte secretion of matrix metallopeptidase 9 (MMP-9), and the endothelial induction of occludin and platelet endothelial cell adhesion molecule (PECAM-1), also known as cluster of differentiation 31 (CD31), in HUVECs [69].

Among the common antioxidants from food with anti-inflammatory activity, resveratrol is one of the most widely studied. Several evidences have demonstrated that resveratrol could improve endothelial function by increasing NO bioavailability and reducing ROS scavenging [70, 71].

Some *in vitro* studies have demonstrated the protective effect of hydroxytyrosol (HT), acting by lowering ICAM-1 and VCAM-1 levels and reducing monocyte adhesion to stimulated endothelium through the involvement of NF-*κ*B [72, 73].

Also, edible marine plants, such as pyrogallol-phloroglucinol-6,6-bieckol, isolated from the edible alga Ecklonia cava, alleviated vascular dysfunction and protected against monocyte-associated endothelial cell death by increasing the phosphorylation of PI3K-AKT and 5′ adenosine monophosphate-activated protein kinase (AMPK) and by inhibiting the proliferation of VSMCs [74].

Today, it is mostly accepted that hyperglycemia-induced oxidative stress plays a central role in endothelial dysfunction by enhancing the production of proinflammatory mediators on vascular cells with some effects that directly impact the vasculature. Therefore, the role of several polyphenols can be studied straightway on the vasculature. Indeed, vascular relaxation impaired by high glucose was observed in rat aortas stimulated with 30 mM glucose and incubated with ellagic acid (EA), a polyphenol found in fruits and nuts [75]. Also, EA displayed antioxidant effect that involved inhibition of ERK1/2 and downregulation of NADPH oxidase 4 (NOX4) showing a vascular-protective effect under diabetic conditions in HAECs [75]. Also, resveratrol was found protective against acute high-glucose-induced damage, whose effect was evaluated in both rat aortic rings and HUVECs [76]. Furthermore, olive oil polyphenols were able to ameliorate endothelial dysfunction induced by high glucose and free fatty acids through modulation of NO and endothelin-1 in endothelial cells [77]. Again, HT, the most abundant polyphenol from olive oil, reduced VCAM-1 and IL-1*β* levels and abated cell proliferation in the vascular wall of streptozotocin-diabetic rats [78].

Among various polyphenols, procyanidins are a class widely found in grapes, cocoa, and fruits. The most abundant source is grapes with many reports about their radical scavenging and biological activities such as regulation of lipid metabolism, inhibition of AGEs formation, and improving role in diabetes and its complications.

Of interest, Jiang and collaborators found that although grape seed proanthocyanidin extracts did not significantly influence the content of NO, they were found helpful in preventing diabetic macrovascular complications, through the decrease of plasma ICAM-1 and VCAM-1 in diabetic rats, as well as in rat aortas evaluated immunohistochemically [79]. Grape seed proanthocyanidin extracts prevented high glucose induced the increase of VCAM-1 and ICAM-1 by PKC and NF-*κ*B inhibition [79].

Interestingly, some recent papers have shown that polyphenols have preventive effect on endothelial dysfunction induced by metabolic stress. Indeed, boysenberry polyphenol was able not only to inhibit endothelial dysfunction in HUVECs by reducing ROS levels and increasing NO production, but the latter was significantly increased also in the aortas of high-fat diet (HFD) mice treated with the polyphenols [80]. Furthermore, the protective effect of anthocyanins purified from boysenberry polyphenols was evaluated in *ex vivo* studies on iliac arteries obtained from mice with dietary obesity. A significant reduction of ROS production was observed together with increase in NO production and suppression of eNOS uncoupling [80]. A similar effect was observed in HUVECs supplemented with mulberry extract, rich in phenols, flavonoids, and anthocyanins [81]. Also, in high-fat diet rabbits, the atherosclerotic lesion was found significantly reduced following treatment with HT [82].

Finally, an important process that has to be taken into account is the endothelial-to-mesenchymal transition (EndMT) which plays a key role in the pathogenesis of several inflammatory diseases. EndMT is characterized by a change in normal endothelial cells that assume the phenotype of mesenchymal cells (fibroblasts and smooth muscle cells). The first step of early EndMT is characterized by downregulation of endothelial markers, such as CD31, vascular endothelial-cadherin, and von Willebrand factor, and upregulation of some early mesenchymal markers such as vimentin, fibronectin, alpha-smooth muscle actin (*α*-SMA). Bioactive compounds may have beneficial effect on markers of endothelial dysfunction by acting to prevent this phenomenon.

An interesting recent paper showed that HT, the major phenolic compound of extra-virgin olive oil and its plasma metabolite HT-3O sulfate (HT-3Os), resulted protective in endothelial cells where IL-1*β* was added to induce EndMT phenotype. The bioactive compound HT-3Os had beneficial effect against IL-1*β*-induced EndMT by restoring let-7, whose level results significantly reduced during EndMT in atherosclerosis [83].

### 2.2. Effects of Polyphenols on the Modulation of Innovative Biomarkers of Endothelial Dysfunction: *In Vitro*, *Ex Vivo*, and Experimental Animal Studies

As mentioned above, besides the known classical biomarkers, in the last years, some studies based on high-throughput technologies have been focused on the investigation of other possible biomarkers of endothelial dysfunction. Certainly, there is a critical need for expedited development of validated biomarkers and their use, in order to improve early diagnostic of endothelial dysfunction and prevent/slow down atherosclerosis and cardiovascular disease [36]. With the advent of omics-based approach and novel technologies, such as microarrays, RNA sequencing, and mass spectrometry, large amounts of data on genes, pathways, and RNAs that are potentially involved in early atherogenesis have been generated.

Recently, the microarray approach to investigate the effect of polyphenols on the expression of some microRNAs has been applied. Changes in miRNA expression were observed in the liver of wild-type (C57B6/J) mice or apoE2/2 mice fed with diets supplemented with different polyphenols. Interestingly, five miRNAs (mmu-miR-291b-5p, mmu-miR-296-5p, mmu-miR-30c-1∗, mmu-miR-467b∗, and mmu-miR-374∗) were modulated by these polyphenols [84]. These miRNAs were localized in pathways involved in adhesion and transendothelial migration. Of note, the expression of miR-296-5p, found to be downregulated in endothelial cells exposed to inflammatory stimulus, was upregulated following treatment with polyphenols [84].

Another miRNA found to be modulated by the antioxidant resveratrol is miR-126. Indeed, this miRNA was effective in preventing oxidative stress in endothelial cells through the activation of PI3K/Akt [85]. Again, resveratrol was found protective on endothelial inflammation by reducing ICAM-1 expression in ECs of thoracic aortas in TNF-*α*-treated wild-type mice but not miR-221/222 knockout mice, thus indicating a mechanism through modulation of miR-221/222 [86]. Another interesting study based on the impact of resveratrol in both progenitor and stem cells showed its effect not only in inducing the expression of the endothelial markers CD31, VE-cadherin, and eNOS but also in reduced miR-21 expression, which in turn decreased Akt phosphorylation [87]. Again, *ex vivo* experiments performed on progenitor cells treated with resveratrol showed better endothelialization of the decellularized vessel [87].

Based on these new findings, it is therefore worth of note to enhance the study of dietary bioactive compounds on ECs by means of omics approaches in order to translate new data into specific targets that can be developed into biomarkers or therapeutics for atherosclerosis.

### 2.3. Effects of Polyphenols on the Modulation of Classical Biomarkers of Endothelial Dysfunction: *In Vivo* Studies

There is not an ideal method for empirical measurement of *in vivo* endothelial function [36]. However, measurements of flow-mediated dilation (FMD) of brachial artery and finger plethysmography (reactive hyperemia index, RHI) have been widely used worldwide. In fact, FMD correlates with the occurrence of future cardiovascular events [88], but it is technically demanding since it requires a high degree of training and experience to ensure accurate and reliable measurements. Nonetheless, FMD is the most used technique for the assessment of endothelial dysfunction, as emerged from the data reviewed below. Considering the evidence implicating inflammation and oxidative stress in atherothrombosis, circulating biomarkers can be grouped according to their role in the disease pathogenesis, namely, endothelial (endothelin, soluble adhesion molecules), oxidative stress (urinary and serum isoprostanes), and inflammatory biomarkers (hsCRP, IL-6).

Several epidemiological studies provided convincing evidence supporting the protective role of polyphenols on cardiovascular prevention. However, in this review, we will focus on randomized clinical trials assessing biomarkers modulated by polyphenols during early phases of endothelial dysfunction. Cocoa is one of the most widely studied polyphenols. Many clinical trials investigated the effects of cocoa on vascular health. In apparently healthy subjects (*n* = 18), the acute consumption of a fatty meal added with cocoa powders with high flavanol contents (918 mg) compared to low content (28 mg) resulted in an improvement of the perturbation in FMD caused by the fatty meal. Although the authors are not able to explain the precise mechanism by which flavanols improve endothelial dysfunction, it apparently does not involve changes in either triglycerides or free fatty acids [89]. Cocoa powder mixed with hazelnuts and green tea (with high polyphenols content, 1817 mg) incorporated into ice cream was able to acutely reduce levels of circulating markers of oxidative stress (serum hydroperoxides and H_2_O_2_) and to significantly improve FMD and RHI in healthy individuals (*n* = 14) [90]. It is worthy of note that in this trial, the choice of the ice cream as source of polyphenols is based on a better stability of polyphenols during long-term storage at low temperature (4°C) [90]. The acute effect of polyphenols contained in cocoa has been tested also in twelve apparently healthy postmenopausal women. The consumption of 84 g of chocolate with high concentration of cocoa (80% cocoa, 395 mg of polyphenols) acutely increased FMD by 2.4% as compared to no changes observed after the intake of chocolate with low concentration of cocoa (35% cocoa, milk chocolate) or white chocolate [91].

Of interest, vascular alterations induced by smoking habits start with the impairment in NO bioavailability further associated with increased adhesion molecule expression and subsequent endothelial dysfunction [92]. Heiss and collaborators observed that flavanol-rich cocoa dose-dependently increased the circulating nitric oxide pool and FMD in 11 young (31 ± 1 years) smokers randomized to take 100 ml cocoa drink with high (176 to 185 mg) or low (<11 mg) flavanol content [93]. In addition, they found that NO-synthase inhibitor (L-NMMA) was able to reverse the observed cocoa-positive effects in a subset of subjects [93]. The longer-term consequence of flavanol-rich cocoa consumption was investigated by the same authors in a similar group of subjects (smokers, aged 27 ± 1 years). The daily consumption of a flavanol-rich cocoa drink (3 × 306 mg flavanols/d) over 7 days resulted in FMD increase at baseline and in sustained FMD improvement at 2 hours after ingestion. Fasted FMD responses increased from 3.7 ± 0.4% on day 1 to 6.6 ± 0.5% (*P* < 0.05) on day 8. FMD returned to 3.3 ± 0.3% after a washout week of cocoa-free diet (day 15) [94].

Loffredo and collaborators tried to explain mechanisms behind the vasodilatation exerted by cocoa acute intake of dark chocolate. They found in smokers that acute ingestion of dark chocolate (>85% of cocoa) was associated with an improvement in FMD via downregulation of NOx-2-related oxidative stress. Urinary isoprostanes significantly decreased, and FMD and NOx significantly increased in smokers but not in healthy subjects [95]. More recently, the same group observed that 60 g of hazelnut and cocoa spread, an Italian product containing a low percentage of cocoa but rich in hazelnut, significantly increased FMD (from 4.3 ± 2.8 to 8.0 ± 3.2%, *P* < 0.001) in 20 “healthy” smokers (30 ± 11 years). Moreover, serum isoprostanes and NOx-2 activation significantly decreased (from 302.8 ± 59.8 to 240.7 ± 90.8 pmol/l (*P* = 0.03) and from 25 ± 4.4 to 22.6 ± 3.2 (*P* = 0.03), respectively) [96].

As smoking habits, arterial hypertension and overweight are important risk factors for endothelial dysfunction. Interestingly, 50 g of chocolate and 70% cocoa/day (2135 mg polyphenols) for 4 weeks are able to significantly improve endothelial function tested through finger plethysmography (RHI 1.94 ± 0.18 to 2.22 ± 0.08, *P* = 0.01) in 20 stage 1 hypertensive subjects with excess body weight (body mass index 25 to 34.9 kg/m^2^). However, no significant changes in biomarkers of inflammation, adhesion molecules, oxidized LDL, and blood pressure were observed [97]. Endothelial dysfunction is regarded as an early manifestation in the pathogenesis of atherothrombosis and vascular complications in patients with type 2 diabetes (T2D). Ingestion of polyphenol-rich chocolate, prior to an oral glucose load, ameliorated oxidative stress, and the adverse effects acutely and transiently induced by hyperglycemia on endothelial function in T2D, as indicated by the improvement of reactive hyperemia peripheral artery tonometry as well as the reduction of adhesion molecules (intercellular adhesion molecule 1, E-selectin and P-selectin glycoprotein ligand 1) and the lower urinary isoprostane excretion [98].

The effect of polyphenols contained in fruits on endothelial function has been widely explored in clinical studies. In healthy subjects, citrus flavonones (orange juice: 128.9 mg to 452.8 mg of flavanones) [99], blueberry [100, 101], and cranberry juice [102] induced an increase of FMD until to 7 hours after consumption in randomized clinical trials. Noteworthy, Rodriguez-Mateos and collaborators observed that the magnitude of vascular effects exerted by blueberries was not changed by kind of food processing (baked or juice), despite kinetic differences. The maximal FMD occurs at 2 h postconsumption of the blueberry-baked buns and at 1 h postconsumption of the blueberry drink [100, 101]. Curtis et al. recently observed, in a bigger sample size study, an improved FMD and arterial stiffness but no effects on pulse wave velocity in 115 overweight/obese adults after 6 months of daily intake of 1 cup of blueberries as compared to 1/2 cup or placebo [103].

In overweight subjects, the acute consumption of a polyphenol-rich fruit (frozen acai pulp: 694 mg of total phenolics) induced an improvement of FMD as well as a reduction in total oxidative status [104]. To our knowledge, a number of studies which found a neutral effect of fruit polyphenols on vascular function in acute as well as chronic administration in overweight subjects are limited [105–107].

In other conditions of endothelial dysfunction, e.g., cigarette smoking or arterial hypertension, fruits' polyphenols (grape or pomegranate juices) enhanced FMD and the pulse wave velocity [108] without improvement in biomarkers of inflammation [109]. Instead, detrimental effects of grape seed polyphenols (1000 mg/day) in combination with vitamin C (500 mg/day) were detected in hypertensive individuals, in which 6 weeks of treatment resulted in a significant increase in mean 24 h blood pressure as compared to polyphenols alone, vitamin C alone, or placebo. In the same subjects, endothelium-dependent and endothelium-independent vasodilation and markers of oxidative damage were not significantly altered by treatments [110]. Conversely, apple-derived polyphenols were able to ameliorate endothelial stiffness measured by pulse wave analysis in 62 overweight subjects with prediabetes (100 mg/dl ≤ fasting plasma glucose ≤ 125 mg/dl) [111].

Polyphenols in the form of chlorogenic acid are highly represented in coffee and tea. The effects of coffee and tea on endothelial function remain controversial. The improvement of endothelial dysfunction assessed by FMD or by RHI was observed in healthy subjects after the ingestion of coffee (containing chlorogenic acid at doses from 89 mg to 412 mg) in several small studies [112–117]. Conversely, unfavorable effect exerted by acute ingestion of coffee as compared to a decaffeinated beverage on the endothelial function tested by FMD in healthy adults was reported in two studies (chlorogenic acid amount not reported) [118, 119]. A longer exposure to coffee (420 mg to 780 mg of chlorogenic acid for 8 weeks) seems to be neutral on endothelial function evaluated by FMD in 74 healthy subjects [120]. On the other hand, in the presence of a cardiovascular risk factor, namely, arterial hypertension (*n* = 19, follow-up of 8 days), the consumption of black tea (150 mg of flavonoids) induced an acute and chronic increase of FMD and of the number of circulating angiogenic cells [121].

Another rich source of dietary polyphenols is extra-virgin olive oil which is also considered to be a pillar of the Mediterranean diet [122, 123]. The consumption of olive oil enriched with phenolic compounds and triterpenes for 3 weeks by healthy subjects affects plasma endothelin-1 concentrations, but other markers of endothelial function, namely, sVCAM1 and sICAM1, resulted unchanged, in healthy subjects [124]. However, at early stage of endothelial dysfunction, the positive effects of olive oil are more evident. In 24 young women with high-normal BP or stage 1 essential hypertension consuming polyphenol-rich olive oil (~30 mg/day of polyphenols) for 2 months, Moreno-Luna and collaborators observed a significant reduction of blood pressure, of markers of oxidation/inflammation, and of ischemia-reactive hyperemia as compared to polyphenol-free olive consumers [125]. A similar finding on blood pressure was found in male with similar clinical characteristics consuming either a phenolic-rich olive leaf extract (136 mg oleuropein, 6 mg HT) or a polyphenol-free control daily for 6 weeks. However markers of inflammation or vascular function were not affected [126].

A meta-analysis provided evidence that olive oil might exert beneficial effects on endothelial function and biomarkers of inflammation in healthy subjects as well as in patients with high risk for cardiovascular events (e.g., diabetes). Olive oil interventions (with daily consumption ranging approximately between 1 mg and 50 mg) resulted in a significantly decrease in hsCRP (mean difference: -0.64 mg/l, 95% confidence interval (CI) -0.96 to -0.31, *P* < 0.0001, *n* = 15) and IL-6 (mean difference: -0.29, 95% CI -0.7 to -0.02, *P* < 0.04, *n* = 7 trials). Values of FMD were found significantly increased in olive oil intervention arms (mean difference: 0.76%, 95% CI 0.27 to 1.24, *P* < 0.002, *n* = 8 trials) [127].

Among polyphenols, resveratrol is one the most widely studied and also the one with the most conflicting clinical results. This regards mainly clinical investigations on weight, blood pressure, and lipid profile. Discrepancies among current evidence might be linked with the differences existing in study design, characteristics of study populations, duration of supplementation, and dosages of resveratrol used. Moreover, the relevant dose of resveratrol is still not clear and it certainly varies depending on the effect being studied [128].

Few studies investigated the effect of resveratrol on endothelial dysfunction. Three different doses of resveratrol (30, 90, and 270 mg) significantly increased FMD (*P* < 0.05) in overweight/obese subjects and in women with untreated borderline hypertension (*n* = 24) as compared to placebo. The authors also observed a linear relationship (*P* < 0.01, *R*^2^ = 0.08) between log10 of plasma resveratrol concentration and acute FMD [129]. van der Made and collaborators, conversely, observed that transresveratrol (150 mg/day for 4 weeks) did not affect fasting or postprandial FMD and plasma biomarkers of endothelial function or inflammation in overweight and slightly obese subjects (*n* = 45) as compared to placebo [130]. A higher dose of transresveratrol (300 mg) acutely and significantly improved FMD in women but not in men with hypertension and baseline endothelial dysfunction [131].

Regarding common dietary resveratrol consumption, it is mainly present in grape berry skins but not in flesh, and this explains the lower amount of the compound in white wine as compared to red wine which traditionally undergoes a higher maceration time [132]. In the nineties, resveratrol has been moved into the limelight for “the French paradox” which describes the association between the consumption of red wine and the relatively low incidence of coronary heart disease in French people despite a high dietary fat intake [133]. In fact, several prospective studies have consistently demonstrated that light-to-moderate red wine consumption (one to two glasses per day) is strongly associated with a lower incidence of cardiovascular events compared with abstinence or occasional alcohol consumption [134–136]. Few clinical studies investigated the effect of red wine on endothelial function with not conclusive results [137–141].

Epidemiological observations need to be validated by conducting controlled human intervention studies. Important aspects of such studies should be the enrolment of well-defined subject populations, standardized outcome measures of oxidative stress, inflammation, and endothelial function. Finally, it will be important to find out the amount of red wine ingestion that is representative of normal healthy dietary intakes.

It is worth of note that supplementation with only one component of the known polyphenols could not be able to replace the benefits of a complex mixture of interacting healthy nutrients as part of a healthy diet. Although several epidemiological studies provided convincing evidence to support the protective role of a “healthy diet” in cardiovascular disease, few controlled clinical trials have been so far conducted. Macready and collaborators investigated in 174 low-fruit and low-vegetable consumers at risk of cardiovascular disease, the effect on vascular function of increasingly doses (from 2 to 6 portions per day) of fruit and vegetables with low or high content in flavonoids as compared to habitual subject intake. A significant increase in endothelium-dependent microvascular reactivity was observed only in men who added to their diet 2 portions/day of fruit and vegetables with high content in flavonoids for 6 weeks. In the same subgroup, hsCRP, E-selectin, and vascular cell adhesion molecule were reduced adding 4 portions/day (at week 12). Moreover, a diet enriched in fruit and vegetables (2/6 portions per day) regardless of flavonoid content was able to mitigate the vascular stiffness measured by pulse wave analysis (PWA) at week 18 [142]. Similar results were observed in subject with I or II grade of arterial hypertension randomized to consume a high-polyphenol diet of six portions of fruit and vegetables (including one portion of berries/day and 50 g of dark chocolate) or a low-polyphenol diet. After 8 weeks, the endothelium-dependent vasodilation assessed by venous plethysmography was significantly improved in high consumers as compared to low polyphenol consumers [143]. The main limitations affecting the comparison and the interpretation of the results obtained from abovementioned studies are the study design as well as dosages and time of supplementation and most of all the methods used for vascular function assessment.

### 2.4. Effects of Polyphenols on the Modulation of Innovative Biomarkers of Endothelial Dysfunction: *In Vivo* Studies

Few clinical studies have investigated the effect of polyphenols on early endothelial dysfunction through the assessment of innovative biomarkers, namely, miRNA, endothelial progenitor cells (EPCs), and extracellular vesicles (EVs).

Rodriguez-Mateos and collaborators recently proposed molecular mechanisms of action of polyphenols from blueberries using nutrigenomic approaches [144]. Daily wild blueberry consumption in healthy subjects led to a dose-dependent improvement of endothelial function in healthy humans, as measured by flow-mediated dilation besides a differential expression (>1.2-fold) of 608 genes and 3 miRNAs: miR126-5p, miR-30c-5p, and miR-181c. The last showed a 13-fold increase in peripheral blood mononuclear cells [144].

Conversely, 4 months of daily intake of cranberry juice did not change vascular responses to reactive hyperemia measured by peripheral arterial tonometry neither circulating EPCs counts in 84 subjects with cardiovascular risk factors. The only effect observed was a decrease in the fraction of EPCs expressing osteocalcin. The authors speculate that, although endothelial function remains unaffected, cranberry juice has a potentially beneficial effect on osteoblastic endothelial progenitor cells, which are linked to the development of atherosclerotic lesions [145].

The effect of a low dose of resveratrol was tested in pathological conditions underlying a late stage of endothelial dysfunction, namely, T2D and arterial hypertension [146]. Microarrays and RT-PCR were used to analyze expression changes in genes and microRNAs involved in the inflammatory response modulated by a 1-year consumption of grape extract enriched or not with resveratrol (8 mg) in individual with diabetes or with hypertension. miRNA involved in the inflammatory response and expressed in PBMCs were highly correlated and significantly altered in the group consuming grape extract enriched with resveratrol [58].

Endothelial EV levels were assessed to evaluate the effects of high-cocoa flavanol drink as compared to a low-cocoa flavanol one in healthy subjects and in patients with coronary artery disease [42]. High-flavanol intervention lowered the levels of CD31^+^/41^−^ and CD144^+^ EVs (-25 and -23%, respectively) but had no effect on CD41^+^ platelet-derived EVs in patients with coronary artery disease along with flow-mediated dilation improvement [42]. However, EV levels were assessed in frozen platelet-free plasma obtained by differential centrifugation. According to some authors, the interpretation of data should take into account the high-speed centrifugation of samples because of the possibility of MV aggregate formation that might affect either the purity and concentration of MVs or their size and biochemical composition [147].

## 3. Carotenoids

### 3.1. Effects of Carotenoids on the Modulation of Classical Biomarkers of Endothelial Dysfunction: *In Vitro*, *Ex Vivo*, and Experimental Animal Studies

Several *in vitro* and *in vivo* animal studies have established a link between carotenoids, oxidative stress, and inflammation suggesting that these bioactive compounds significantly contribute to the redox balance protection. Of note, carotenoids inhibit cell adhesion and migration to the endothelium by blocking the activation of NF-*κ*B, CD14, and Toll-like receptor 4 (TLR4) expressions and TNF-*α*-induced ICAM-1 and VCAM-1 expressions in both vein and arterial endothelial cells [148, 149]. Furthermore, carotenoids have been found efficient in maintaining endothelial NO bioavailability, suggesting their ability to preserve endothelial function and more in general vascular health [150].

In this regard, we recently demonstrated that both *β*-carotene and lycopene had a protective effect in HUVECs, following TNF-*α*-induced inflammation. In detail, this was associated with a significant decrease in ROS and nitro-tyrosine generation, as well as increased NO bioavailability. Moreover, downregulation of NF-*κ*B-dependent adhesion molecule expression and monocyte-HUVEC interaction was found following pretreatment with carotenoids [16]. More recently, we investigated the effect of *β*-carotene and lycopene in HUVECs obtained from the umbilical cords of women with gestational diabetes (GD). Interestingly, we found that both *β*-carotene and lycopene could attenuate endothelial dysfunction in GD-HUVECs, demonstrated by reduced TNF-*α*-induced nuclear translocation of NF-*κ*B, lowered VCAM-1 and ICAM-1 total expression levels and membrane exposure, and significantly reduced peroxynitrite levels [15]. These results suggest a new mechanism of action of carotenoids which display vascular protective action in diabetic condition by supporting the redox balance and preserving NO bioavailability.

However, it should be taken into account that following stimulation of HUVECs with TNF-*α*, NO rapidly reacts with O_2_^−^ to form the stable oxidant ONOO^−^ resulting in reduced vascular relaxation and contributing to the upregulation of NF-*κ*B-dependent cellular response. Of note, the main effect of antioxidant molecules, such as carotenoids, on the biological function of NO is in part due to the direct removal of O_2_^−^ [151].

Another effect of carotenoids is the regulation of ET-1 expression, a potent vasoconstrictor synthesized by endothelial cells that plays a crucial role in the pathophysiology of CVD. In detail, it was found that lycopene inhibits cyclin strain-induced ET-1 expression through the suppression of ROS generation and induction of heme oxygenase-1 in HUVECs [152].

Again, in H_2_O_2_-treated human vascular endothelial cells (ECV304 cells), lycopene prevented oxidative injury and decreased dose-dependently malondialdehyde (MDA) levels [153].

Of note, considering the strong antioxidant effects of lycopene *in vitro*, its administration *in vivo* has received attention [154]. It was found that male Wistar rats fed for 31 days with lycopene dissolved in olive oil had reduced lipid peroxides and increased glutathione levels [155]. Similar results were obtained in female Wistar rats fed with lycopene for 2 weeks where the activity of glutathione reductase, glutathione peroxidase, and superoxide dismutase (SOD) was found significantly improved [156]. In a hyperhomocysteinemia rat model, lycopene showed antiatherogenic effects by reducing serum markers of inflammation such as VCAM-1, monocyte chemoattractant protein 1 (MCP-1), and IL-8 [157]. Chronic lycopene treatment can ameliorate endothelial dysfunction in streptozotocin-induced diabetic rats and it might be useful in preventing the diabetic vascular complications associated with endothelial dysfunction [158].

The effect of lycopene on atherosclerosis induced by a high-fat diet in adult male New Zealand white rabbits was compared to fluvastatin [159]. Lycopene was more beneficial than fluvastatin in reducing levels of total cholesterol, total triacylglycerol, and LDL cholesterol [159].

Only two studies conducted in rabbits demonstrated that lycopene had no effect in improving the aorta lesions. One study conducted in male Watanabe heritable hyperlipidemic rabbits demonstrated that lycopene had no effects on cholesterol and triacylglycerol levels in plasma and on aortic atherosclerosis [160]. The other study was carried out in New Zealand white rabbits and, although it was found reduced LDL cholesterol serum levels and lower cholesteryl ester in the aorta of lycopene-treated group, no significant improvements in the aorta lesions were detected [161].

All together, these results suggest that lycopene administered alone or in combination with other molecules of nutritional interest has a protective effect on endothelial cells by preventing oxidative injury and improving cardiovascular-related beneficial effects.

Another interesting study suggests that *β*-carotene significantly reduced intracellular accumulation of ROS in RAW264.7 cells stimulated with lipopolysaccharide (LPS), thus showing anti-inflammatory activity as a potential inhibitor of NF-*κ*B activation [162].

Moreover, *β*-carotene was found beneficial in regulating the expression of eNOS and adhesion molecules via the Ca^2+^/calmodulin-dependent protein kinase II (CaMKII) pathway activation in an *in vitro* model of endothelial dysfunction induced by IL-1*β* [163].

Interestingly, the protective effect of *β*-carotene was demonstrated by *in vivo* studies. Indeed, annatto extract or *β*-carotene supplemented to the diet of rats for 7 days decreased ROS production and increased mRNA levels of superoxide dismutase (SOD), catalase (CAT), p22(phox), and p47(phox), which are components of the innate antioxidant defence [164].

These results suggest that *β*-carotene has a notable anti-inflammatory activity by protecting the endothelium against oxidative stress.

Another carotenoid with antioxidant activity and beneficial effect on endothelial function preservation is astaxanthin, a xanthophyll with remarkable cell membrane potential. This molecule is able to neutralize free radicals by either accepting or donating electrons [165]. Also, astaxanthin was found efficient in ameliorating diabetic endothelial dysfunction by inhibiting the ox-LDL-eNOS pathway [166].

Evidences indicate that carotenoids might be involved in other molecular pathways, such as those related to cell proliferation, acting at Akt, tyrosine kinases, MAPK, and growth factor signaling cascades [167]. In addition, further mechanisms such as modulation of lipid metabolism have been suggested as significant effect on endothelial function [168].

Therefore, carotenoids may be considered promising antioxidant modulators of endothelial response to inflammatory stimuli, suggesting that an adequate carotenoid consumption could represent a useful tool for the prevention of oxidative stress and diabetes cardiovascular complications.

### 3.2. Effects of Carotenoids on the Modulation of Classical Biomarkers of Endothelial Dysfunction: *In Vivo* Studies

The major dietary sources of carotenoids are pigmented fruits, juices, and vegetables. In particular, yellow vegetables and fruits provide most of the *β*-carotene and *α*-carotene, orange fruits provide *α*-cryptoxanthin, dark green vegetables provide lutein, and tomatoes/tomato products provide lycopene [169]. Processing and storage of food leading to enzymatic or nonenzymatic oxidation of the highly unsaturated carotenoid molecules are the principal causes of carotenoid loss of function [170]. In general, common household cooking methods do not alter carotenoid content of foods, but extreme heat can result in oxidative destruction of these antioxidant compounds [171].

A number of epidemiological studies support the notion that elevated dietary carotenoid intake is associated with high circulating levels of their active compound and with the prevention of cardiovascular disease [172]. Among all carotenoids, lycopene has been researched extensively in recent years for its role in the prevention of total and cause-specific mortality as well as to cardiometabolic risk factors [173]. It is one of the most effective antioxidant carotenoids with twice the antioxidant activity when compared to *β*-carotene [174, 175].

The evidence from human intervention trials on the efficacy of tomato products or lycopene supplementation on endothelial function assessed by FMD and pulse wave velocity (PWV) has been meta-analyzed by Cheng and collaborators [176]. Six studies including 233 participants evaluated the impact of tomato supplementation on FMD. Healthy subjects, healthy postmenopausal women, and ultramarathon runners were involved in these studies. Although acute intervention did not improve FMD, short-term (~1 week) tomato supplementation significantly increased FMD by 2.53% (95% CI 0.56 to 4.50; *P* = 0.01). Among these studies, only Stangl and colleagues failed to show improvement of endothelial function acutely and after 7 days of buttered roll with tomato consumption in postmenopausal women [177].

Forearm responses to intra-arterial infusions of acetylcholine (endothelium-dependent vasodilatation), sodium nitroprusside (endothelium-independent vasodilatation), and NG-monomethyl-L-arginine (basal nitric oxide synthase activity) were measured using venous plethysmography in 36 patients with cardiovascular disease and 36 healthy subjects. Lycopene supplementation (7 mg) was able to improve the endothelial-dependent vasodilation in patients with cardiovascular disease, but not in aged-matched healthy controls [178]. Aortic PWV and augmentation index gave no statistically significant differences in all study participants. The authors speculate that lycopene may exert its action especially on smaller vessels, such as resistance arteries, rather than larger vessels, since measured arterial stiffness remained unaltered [178].

Regarding circulating markers of endothelial function, Kim and collaborators reported a positive outcome among 126 individuals equally divided into 3 groups who consumed either 6, 15 mg lycopene/day or placebo [179]. Endothelial function assessed by reactive hyperemia peripheral arterial tonometry (RH-PAT) and biomarkers (sICAM-1, sVCAM-1, and hsCRP) assessed after 8 weeks of treatment were significantly modified by the higher lycopene supplement [179].

Conversely, lycopene supplementation was ineffective at reducing sVCAM-1 and sICAM-1 in young healthy subject (*n* = 27; 80 mg/day of lycopene for 1 week) [180] as well as in moderately overweight, healthy, middle-aged individuals (*n* = 79; 10 mg/day of lycopene for 12 weeks) [181].

Tomato product consumption (94 g of tomato paste, 27 mg of lycopene) acutely reduced oxidative stress induced by postprandial lipemia and the associated inflammatory reaction (IL-6) in normal weight participants (*n* = 25), although FMD was not affected [182].

Most of the studies evaluating the effect of carotenoids on the early endothelial dysfunction were focused on lycopene which showed encouraging but not conclusive results in terms of vascular benefit due to differences in concentration and timing of administration adopted in the study protocols.

### 3.3. Potential Strategies to Establish Innovative Biomarkers of Endothelial Dysfunction Modulated by Carotenoids

To date, the modulation of the known classical biomarkers indicates a positive impact of dietary carotenoid ingestion in the reduction of risk of inflammation. Many studies showed an inverse relationship between carotenoids and development of endothelial dysfunction by reducing oxidative stress through changes of the classical biomarkers described above. However, although high-throughput technologies have been applied to polyphenols, no studies based on these novel approaches have been performed on carotenoids so far. Therefore, the role of carotenoids that counteract oxidative stress and promote healthy aging is worthy of further investigation. Omics information may be collected from endothelial cells pretreated with carotenoids as well as from samples of individuals treated with a diet rich of these bioactive molecules. These measurements, along with standard tests, may allow to gain insight of the role of carotenoids in a diet and expect to better assist in health care in many ways including early and accurate diagnosis, monitoring disease progression and dietary intervention, targeted dietary treatments, and disease prevention. Last but not least, omics technologies may provide novel dietary intervention for personalized nutrition approach leading to a deeper understanding of interindividual variability in response to diet.

## 4. Conclusions and Future Perspectives

In this review, we report the significance of polyphenols and carotenoids as a source of beneficial compounds for human health with focus on their role in the prevention of endothelial dysfunction. However, further clinical trials are needed for defining dosages and time of exposure of polyphenols and carotenoids able to bring a concrete benefit to the vascular function.

Of note, an important point to be raised is that polyphenol compounds have poor bioavailability due to slight absorption in the gut, intestine, and colon, thus weaken their health-promoting effect [183]. Likewise, carotenoids have sensitivity against environmental and process stresses, low-water solubility, and low bioavailability [184].

Recently, different strategies have been applied to improve their absorption and bioavailability. Interestingly, nanoparticles based on different chitosan derivatives and poly(lactic-co-glycolic acid) (PLGA), a widely approved synthetic biocompatible and biodegradable polymer, were successfully used to encapsulate polyphenols from cherry extracts, thus improving their antioxidant and anti-inflammatory activity [185–187].

Interestingly, an *in vitro* coculture cell model that combines intestinal absorption with changes in endothelial function has been generated to test the effect of resveratrol. The crosstalk between gut epithelial and endothelial cells implicates the activation of immune and inflammation signaling pathways, which represent key modulators of endothelial dysfunction [188]. The methylated analogue of the polyphenol resveratrol, the 2,3′,4,5′-tetramethoxystilbene (TMS), a potent antioxidant, has been encapsulated in liposome to improve its bioavailability. This delivery method has been demonstrated more efficient in restoring the endothelium-dependent dilation via NO than TMS solution alone [189].

Lately, the synergistic effect of current antihyperglycemic drugs already in use together with natural bioactive compounds has been tested as a complementary therapeutic approach to improve the bioactivities and bioavailability of these compounds in order to treat inflamed ECs and prevent cardiovascular events in diabetic patients [190, 191].

Besides novel approaches to improve polyphenol bioavailability, further studies on sensitive measures of early effects of a dietary intervention are of great relevance. Innovative strategies in which interrelated biological aspects are studied are crucial to fully understand the role of environmental factors, including nutrition, to improve individual's well-being.

**Figure 2:**
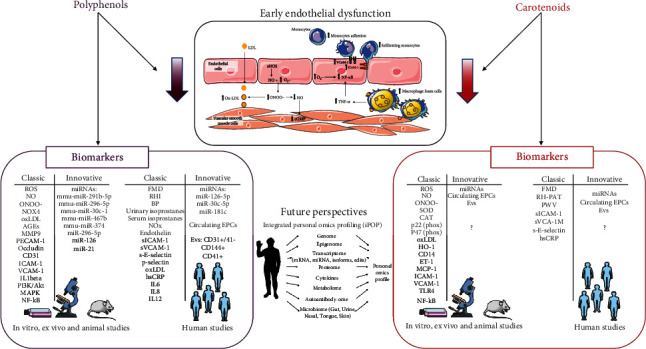
Classical and innovative biomarkers modulated by polyphenols and carotenoids in early endothelial dysfunction. *Part of the figure is adapted from Di Pietro N et al. JSM Atherosclerosis, 2016* [12]. Both polyphenols and carotenoids have been shown to significantly reduce all the key events of early endothelial dysfunction through their antioxidant and anti-inflammatory actions, as described in detail in the figure text. This is proven by their ability in modulating several classic and some innovative biomarkers here summarized. Among the future innovative perspectives, integrated personal omics profiling (iPOP) will potentially allow to obtain personalized prevention and nutritional food intervention through the intricate and mutual interaction of omics technology (genome, epigenome, transcriptome, proteome, cytokines, metabolome, autoantibody-ome, and microbiome). eNOS: endothelial nitric oxide synthase; NO: nitric oxide; O_2_^−^: superoxide anion; ONOO^−^: peroxynitrite; cGMP: cyclic guanosine monophosphate; LDL: low-density lipoprotein; ox-LDL: oxidized low-density lipoprotein; TNF-*α*: tumor necrosis factor alpha; NF-*κ*B: nuclear factor kappa-light-chain-enhancer of activated B cells; ICAM-1: intercellular adhesion molecule 1; VCAM-1: vascular cell adhesion molecule 1; ROS: reactive oxygen species; NOX4: NADPH oxidase 4; AGEs: advanced glycation end-products; MMP9: matrix metallopeptidase 9; PECAM-1: platelet endothelial cell adhesion molecule; clusters of differentiation: CD31, CD31+/41-, CD144+, CD41+, and CD14; interleukins: IL1beta, IL6, IL8, and IL12; PI3K/Akt: phosphoinositide 3-kinases/protein kinase B; MAPK: mitogen-activated protein kinase; FMD: flow-mediated dilation; RHI: reactive hyperemia index; BP: blood pressure; RH-PAT: reactive hyperemia peripheral arterial tonometry; PWV: pulse wave velocity; NOx: nitrogen oxides; sICAM-1: soluble ICAM-1; sVCAM-1: soluble VCAM1; sE-selectin: soluble E-selectin; hsCRP: high-sensitivity C-reactive protein; EPCs: endothelial progenitor cells; EVs: extracellular vesicles; SOD: superoxide dismutase; CAT: catalase; ET-1: endothelin-1; MCP-1: monocyte chemoattractant protein 1; TLR4: Toll-like receptor 4.

Here, we have reported several evidences demonstrating that a diet rich in polyphenols and carotenoids may have anti-inflammatory effects by modulating “classical” and some “innovative” biomarkers (see Figure 2). Although the application of systems biology to the study of early endothelial dysfunction is quite premature, these findings are already contributing to novel understandings into the development of endothelial dysfunction and atherosclerosis. However, to provide better scientific evidences of the effects on human health of food rich in antioxidant molecules, further nutrigenomics studies based on the complete integration of various omics technologies, including genomics, transcriptomics, proteomics, metabolomics, and lipidomics, together with bioinformatics approaches, should be encouraged. Worth of interest are proteomics and metabolomics considering their closer link to the phenotype, thus providing more detailed measures of a physiological state. In particular, food metabolomics is of great relevance not only to acquire comprehensive and detailed molecular composition of food but also to collect data of small-molecule intermediates and products of cellular metabolism during a particular treatment or diet. Indeed, metabolite information can be integrated with biological phenotypes and can improve in-depth understanding of pathways in response to dietary polyphenol and carotenoid supplementation.

It should be emphasized that these innovative technologies make it possible to measure the nutritional phenotype carefully in individuals at various stages of disease, thus monitoring changes due to food intervention. Therefore, the intricate and reciprocal interaction of omics technologies may allow to achieve the so-called integrated personalized omics profiling (iPOP), which can be translated into personalized disease prevention and effective treatment plans [48, 53, 54]. The emerging iPOP technology may provide novel insights into health and disease states of individuals that aim not only to the treatment but eventually to counteract endothelial dysfunction, a critical target in preventing the progression of atherosclerosis and cardiovascular disease.
